# Characterization of adolescents with functional respiratory disorders and prior history of SARS-CoV-2

**DOI:** 10.1186/s40348-023-00165-3

**Published:** 2023-09-12

**Authors:** Sebastian Felix Nepomuk Bode, Anja Schwender, Monika Toth, Christine Kaeppler-Schorn, Ute Siebeneich, Joachim Freihorst, Ales Janda, Dorit Fabricius

**Affiliations:** Department of Pediatrics and Adolescent Medicine, Ulm University Medical Center, Ulm University, Eythstrasse 24, Ulm, 89075 Germany

**Keywords:** Long-COVID syndrome, Functional respiratory disorder, Dysfunctional breathing, Hyperventilation, Exercise-induced laryngeal obstruction

## Abstract

**Background:**

The SARS-CoV-2 pandemic has caused significant pulmonary morbidity and mortality in the adult population. Children and adolescents typically show milder symptoms; however, a relevant proportion of them report persistent pulmonary symptoms even after mild SARS-CoV-2 infection. Functional respiratory disorders may be relevant differential diagnoses of persistent dyspnea. This study aims at characterizing functional respiratory disorders that may arise after SARS-CoV-2 infection regarding their clinical presentation and pulmonary function tests as well as gaining insights into the clinical course after initiation of appropriate therapy.

**Methods:**

This study retrospectively identified all patients referred to an outpatient clinic for pediatric pulmonology with functional respiratory disorders manifesting after proven SARS-CoV-2 infection between January 1, 2022, and October 31, 2022. Clinical history, thorough clinical examination regarding breathing patterns, and pulmonary function tests (PFTs) were taken into consideration to diagnose functional respiratory disorders.

**Results:**

Twenty-five patients (44% female) with mean (m) age = 12.73 years (SD ± 1.86) who showed distinctive features of functional respiratory disorders after SARS-CoV-2 infection (onset at *m* = 4.15 (± 4.24) weeks after infection) were identified. Eleven patients showed thoracic dominant breathing with insufficient ventilation, and 4 patients mainly had symptoms of inducible laryngeal obstruction. The rest (*n* = 10) showed overlap of these two etiologies. Most patients had a flattened inspiratory curve on spirometry and slightly elevated residual volume on body plethysmography, but values of PFTs were normal before and after standardized treadmill exercise testing. Patients were educated about the benign nature of the condition and were offered rebreathing training. All patients with follow-up (*n* = 5) showed normalization of the breathing pattern within 3 months.

**Conclusions:**

Functional respiratory disorders are important differential diagnoses in persisting post-SARS-CoV-2 dyspnea in adolescents. A combination of clinical history, detailed examination of breathing patterns, and pulmonary function tests are helpful to correctly diagnose these conditions. Reassurance and rebreathing training are the mainstay of the therapy. The clinical course is favorable.

## Background

The SARS-CoV-2 pandemic caused challenges for both the general public and healthcare services. Pulmonary morbidity and mortality, especially of earlier SARS-CoV-2 variants, have been of significant concern in the adult population. In children and adolescents fortunately, symptom burden and pulmonary manifestations during acute SARS-CoV-2 infection are less severe. Typical symptoms include upper airway affections or gastrointestinal symptoms [1]. Many children and adolescents are even asymptomatic during acute infection [2], and in both short- and long-term follow-up, pulmonary function tests (PFTs) are normal in most cases [3, 4]. Severe pulmonary involvement with long-term sequelae after SARS-CoV-2 infection, especially with the later variants, is rare in children and adolescents [5].

Persistent symptoms after SARS-CoV-2 infection have been described as long-COVID (persistent symptoms up to 3 months after infection) or post-COVID (persistent symptoms longer than 3 months after infection) syndromes [6]. These terms encompass a variety of symptoms and may include fatigue, reduced exercise capacity, (exercise-induced) dyspnea, palpitations, etc. The pathophysiology of these conditions is not fully understood, and some authors have suggested neurological involvement [7, 8], changes in central chemosensitivity [9], diaphragm muscle weakness [10], or autonomous dysfunction [11] after SARS-CoV-2 infection as possible causes. While long-COVID might be a significant problem, especially in teenage girls [6], it is essential to exclude possible differential diagnoses [12]. As up to 50% of children and adolescents have been reported to suffer from persistent dyspnea after SARS-CoV-2 infection, this warrants considerations regarding pulmonary conditions or other causes.

Cardiopulmonary exercise testing is considered the gold standard for measuring exercise capacity and to identify possible pulmonary long-term sequelae after SARS-CoV-2 [13]. Chronotropic incompetence and abnormal peripheral oxygen extraction/use have been identified as contributing factors in patients with long-COVID 3 months after the initial infection, and impaired exercise capacity was demonstrated in these patients [14]. Reduced physical fitness as only cause for persisting symptoms, even after mild SARS-CoV-2 infection, has been described as well [15, 16]. There is also evidence that some patients might show postinfectious bronchial hyperreactivity, even months after SARS-CoV-2 infection [4]. These subjects typically present with dry cough, worsened on exercise, and might benefit from inhaled corticosteroids [17]. Additionally preexistent asthma might worsen and only be diagnosed as symptoms emerge after airway infection [17].

In many patients though, there is a discrepancy between objective and subjective pulmonary symptoms as well as quality-of-life measures [6, 18]. Dysfunctional breathing, also termed functional respiratory disorders, with hyperventilation [19–21] as well as without hyperventilation [21, 22], is possible causes for persistent dyspnea after SARS-CoV-2 infection in adults, as well as in children and adolescents [3, 4]. Dysfunctional respiratory symptoms can occur after infections, psychosocial stress, and other triggers [23]. Different forms have been identified, and different classifications have been used. Manifestations include persistent hyperventilation and inducible laryngeal obstruction (ILO), which may manifest while resting or on exercise [24]. Dysbalanced muscular effort with increased thoracic muscular movement and reduction of diaphragmal action can lead to a thoracic dominant breathing with insufficient ventilation. Functional respiratory disorders are characterized by sudden onset of symptoms (within seconds) without apparent trigger, disappearance of symptoms while sleeping, normal oxygen saturation, and, in many cases, normal clinical examination and normal pulmonary function tests [25]. Some of the conditions present with mostly inspiratory dyspnea; ILO can even present with stridor [25]. Symptom onset immediately or very shortly after start of physical exercise is commonly described, but symptoms can occur while resting [26]. Clinical characteristics, pulmonary function tests, and clinical course of functional respiratory disorders of children and adolescents after SARS-CoV-2 infection are not well understood.

This study aimed to retrospectively characterize whether functional respiratory breathing patterns in patients referred for evaluation of persistent dyspnea or cough after SARS-CoV-2 infection while resting or on exercise were present in patients presented to our outpatient clinic for pediatric pulmonology between January 1st and October 31st, 2022. We wanted to determine whether patients might show specific abnormalities in history, clinical examination, and pulmonary function tests after SARS-CoV-2 infection. We sought to gain insights into the clinical course of the patients after initiation of appropriate therapy.

## Methods

All patients that were evaluated for dyspnea or cough that persisted after a SARS-CoV-2 infection at our outpatient clinic for pediatric pulmonology between January 1st and October 31th, 2022, were retrospectively identified through electronic patient records. SARS-CoV-2 infection had to be proven by either antigen or PCR testing. History and clinical examination; pulmonary function tests including spirometry, body plethysmography, and standardized treadmill exercise tests; and radiological and laboratory examinations were considered. Pulmonary function tests while resting, after treadmill exercise, and in further course after therapy initiation were included in the analysis. Patients were monitored regarding their breathing pattern both while resting, while exercising, and after exercise. Patients were excluded if their symptoms could be explained by another diagnosis, e.g., cardiac diagnosis, pneumonia, asthma, and when patients did not show evidence of functional respiratory disorders. Patients that did not report pulmonary symptoms but only other symptoms congruent with long-COVID were not included. Patients who just had reduced physical fitness, only evidence of chronic fatigue syndrome, or postexercise malaise were also excluded.

Recruitment period was not extended beyond October 2022 as (a) most adolescents in Germany were seropositive for SARS-CoV-2 at this time and reinfections might cause less symptoms, and (b) most importantly, mandatory SARS-CoV-2 testing in schools was abolished in Germany in the beginning of October 2022. Afterwards, SARS-CoV-2 was not tested regularly anymore making the association of dysfunctional breathing patterns with SARS-CoV-2 infection difficult.

### Ethics

The study was approved by the Ulm University Ethics Committee (permit no: 292/22). As this was a retrospective non-interventional study, the need for informed consent was waived.

### Statistical analysis

Categorial data as frequencies, means (m), standard deviation (SD), median, minimum (min), maximum (max), and quartiles are reported. Mann–Whitney *U*-tests/*T*-tests for parallel groups were used to determine differences between initial pulmonary function tests, PFTs after treadmill exercise, and PFTs on follow-up. Statistical analysis was performed with GraphPad Prism (Version 7.01, GraphPad Software, La Jolla, CA, USA, www.graphpad.com).

## Results

Between January 1st and October 31st, 2022, we identified 25 patients (mean age = 12.73 years (± 1.86 years, min. 8–max 15 years), 11 females (44%), 14 males), who showed distinctive features of functional respiratory disorders after a SARS-CoV-2 infection. All SARS-CoV-2 infections had been mild, and no patients had been hospitalized during acute infection. Upper respiratory symptoms as rhinitis were reported by 10 patients (40%) and lower respiratory symptoms as cough by 11 patients (44%). General malaise, headaches, and fever were present in 12 patients (48%) and gastrointestinal symptoms in two (8%). Two patients (8%) were asymptomatic, and four (16%) could not recall if they had any symptoms. Nine patients (36%) had been diagnosed with asthma before, and five (20%) had allergic rhinoconjunctivitis. Dysfunctional respiratory symptoms were noted at a mean (m) = 4.15 (± 4.24, min 1–max. 20) weeks after infection. All 25 patients (100%) reported respiratory symptoms while exercising and 8 (32%) additionally while resting. Details on dyspnea symptoms can be found in Table 1. Four (16%) patients mainly had symptoms of inducible laryngeal obstruction, and 11 (44%) patients showed typical thoracic dominant breathing with insufficient ventilation. The rest (*n* = 10, 40%) showed overlap of these two etiologies. Two patients (8%) reported emotional stress as trigger, whereas one patient (4%) was symptomatic while playing the trumpet.

**Table 1:**
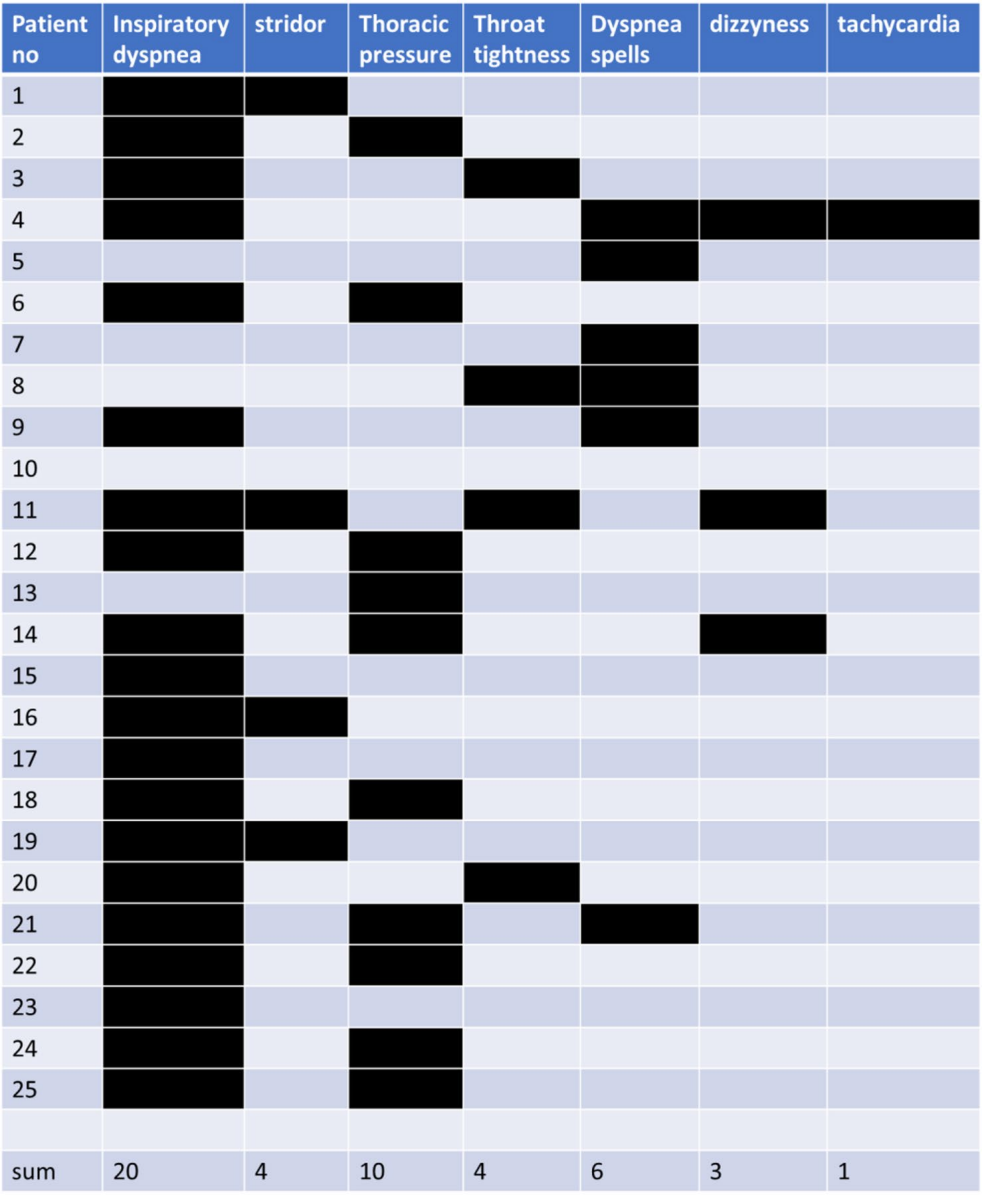
Details on symptoms reported by *n* = 25 patients with functional respiratory disorder patterns after SARS-CoV-2 infection. Patient 10 reported reduced exercise capacity and could not further describe dyspnea symptoms

**Fig. 1:**
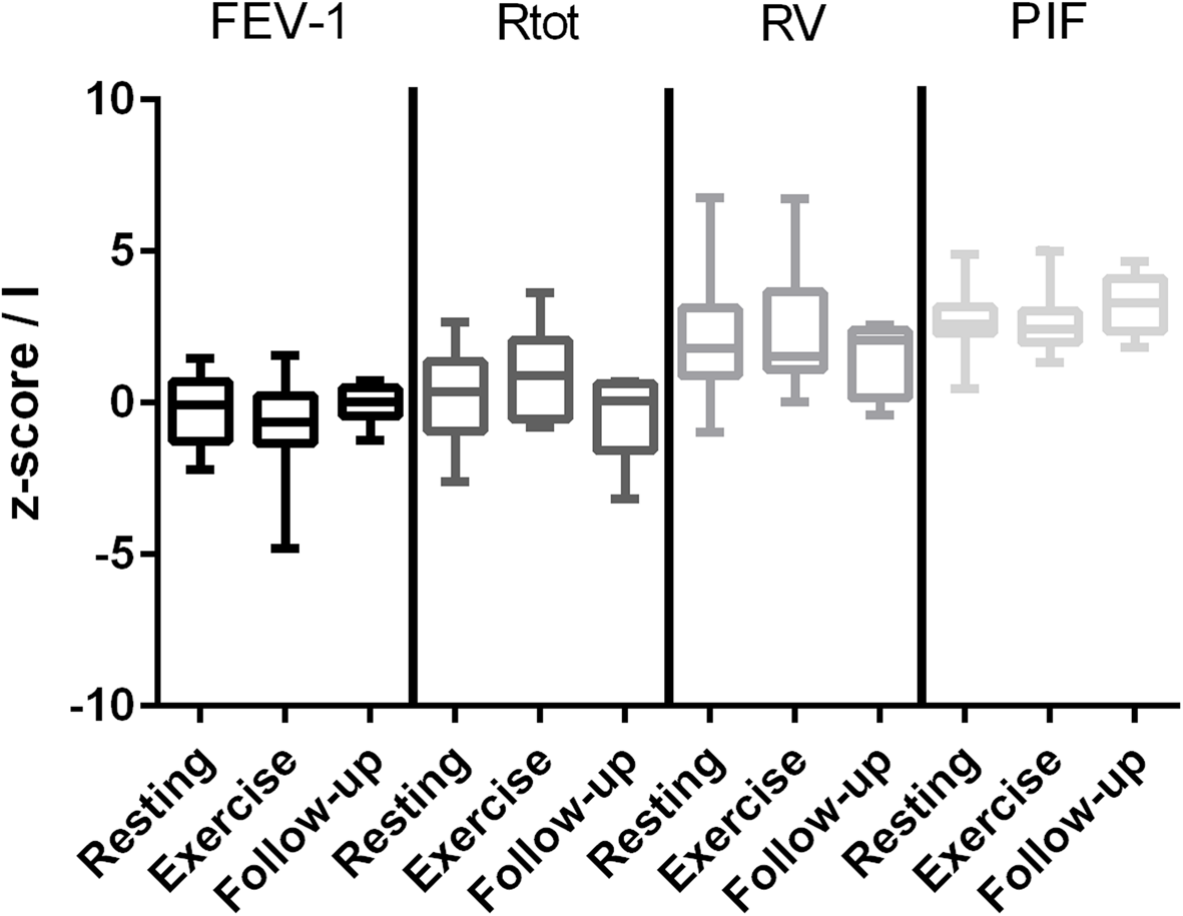
No differences in z-scores of forced expiratory volume in 1 s (FEV-1), resistance (Rtot), residual volume (RV), and peak inspiratory flow (PIF, in liters) while resting (*n* = 25) and after standardized treadmill exercise (*n* = 20) as well as during follow-up (*n* = 5). Mean, 25–75% quartiles and 95% confidence interval depicted

**Fig. 2:**
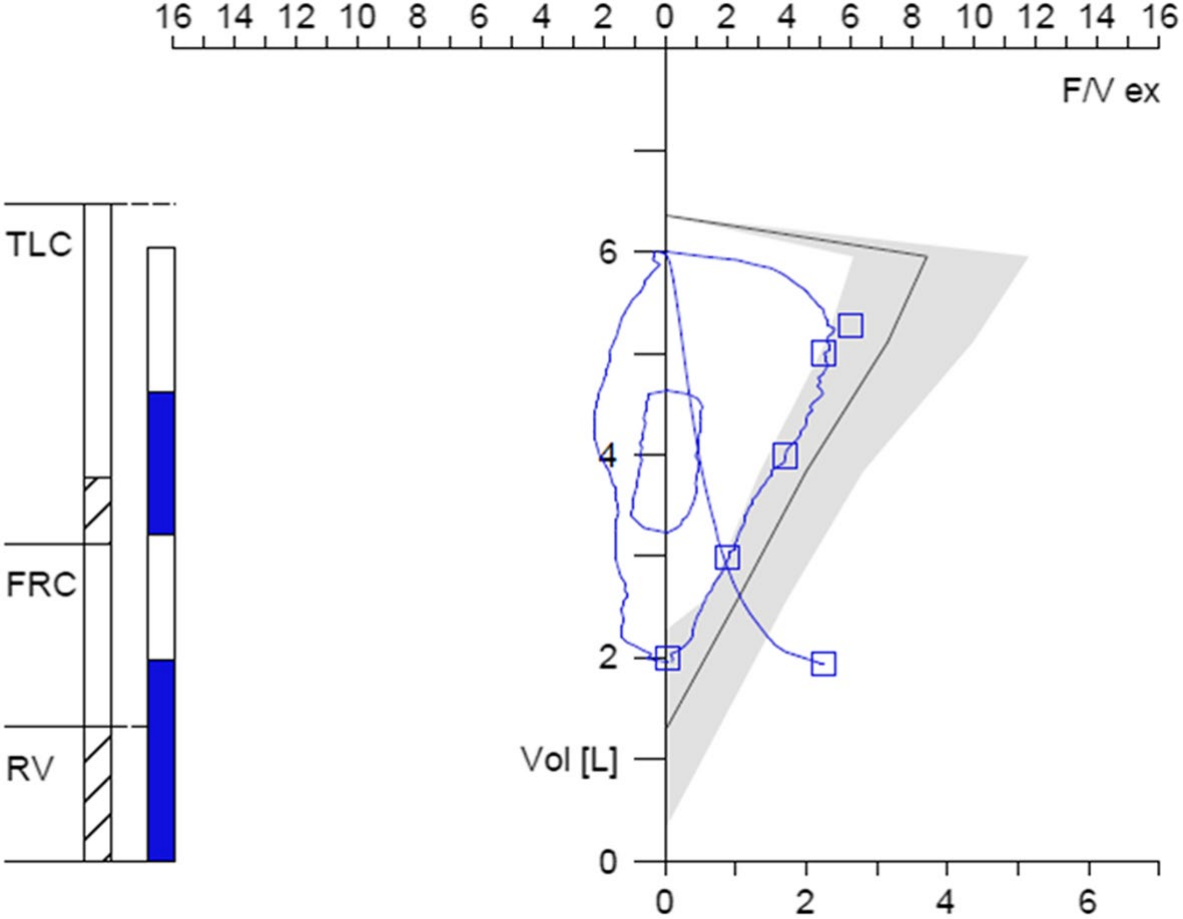
Mildly increased residual volume and slightly flattened inspiratory curve on spirometry in a 14-year-old boy with functional respiratory symptoms of thoracic dominant breathing (with insufficient ventilation) 8 weeks after SARS-CoV-2 infection. FRC, functional residual capacity; RV, residual volume; TLC, total lung capacity

Spirometry and body plethysmography were performed in all patients. Twenty patients underwent standardized treadmill exercise for 6 min and performed spirometry as well as body plethysmography again. All patients that underwent exercise testing clearly showed respiratory symptoms and breathing patterns consistent with a functional respiratory disorder, and all showed normal oxygen saturation > 95% during the exercise test. Z-scores of forced expiratory volume in 1 s (FEV-1), total resistance (Rtot), residual volume (RV), peak expiratory flow (PEF), and peak inspiratory flow (PIF, in liters) were recorded while resting and after exercise (Tables 1 and 2). Spirometry and body plethysmography parameters all were within normal ranges while resting, after 6 min treadmill exercise, and on follow-up (Fig. 1, Table 2). *N* = 20 patients showed a flattened inspiratory curve on spirometry (for an example, see Fig. 2), 16 had an elevated residual volume over the upper limit of normal (for an example, see Fig. 2), but the difference was not significant. Two patients had normal pulmonary function tests.

**Table 2:** No differences in z-scores of forced expiratory volume in 1 s (FEV-1z), resistance (Rtotz), residual volume (RVz), and peak inspiratory flow (PIF, in liters) while resting and after standardized exercise as well as during follow-up in 25 patients (5 patients in follow-up) with functional respiratory disorders after SARS-CoV-2 infection

	**Resting**	**Exercise**	**Follow-up**
FEV-1z (m, ± SD)	− 0.28 (1.05)	− 0.82 (1.38)	− 0.04 (0.67)
Rtotz (m, ± SD)	0.27 (1.44)	1.01 (1.21)	− 0.36 (1.6)
RVz (m, ± SD)	2.28 (2.12)	2.34 (1.85)	1.43 (1.24)
PIF (m, ± SD)	2.75 (1.01)	2.57 (0.88)	3.26 (0.99)

All patients and their parents were educated about the benign nature of the condition and reassured that no pulmonary pathology was present (other pathologies had been ruled out in these patients as described in the in-/exclusion criteria). They were instructed regarding their breathing pattern and possible breathing exercises depending on the functional respiratory disorder that was identified. All were offered physiotherapy-led rebreathing training in an outpatient setting. Five patients have been followed up after a mean of 3 months, and all reported major clinical improvement and showed improved PFTs.

## Discussion

We report one of the first cohorts of adolescents with functional respiratory disorders after mild SARS-CoV-2 infection. Our data show normal pulmonary function tests both while resting and after standardized treadmill exercise. Some patients showed a slightly increased residual volume and a flattened inspiratory curve with reduced peak inspiratory flow. Functional respiratory disorders commonly emerge after mild SARS-CoV-2 infections [21]. Spirometry and body plethysmography typically show no clear pattern in functional respiratory disorders [27], but increased RV and normal FEV-1 have been reported in other pediatric studies in some forms of functional respiratory disorders [17]. Motiejunaite et al. reported on hyperventilation on incremental exercise testing in adults [19], who mostly had been treated as inpatients during acute SARS-CoV-2 infection, and 18% had required mechanical ventilation. Three months after infection, 40% had a reduced carbon monoxide diffusion capacity, 65% of participants had pathological changes on CT, and one-third showed hyperventilation on exercise. Skjorte et al. reported functional respiratory disorders in 11/156 adults [20], and Loew et al. report on a cohort of more than 50 adults with hyperventilation, periodic sigh breathing, and mixed dysfunctional breathing types after SARS-CoV-2 infection [21]. Cardiopulmonary exercise testing (CPET) was normal in almost 1/3 of patients, but functional respiratory disorders were diagnosed with CPET in the remaining 70% [22, 28]. Our data is therefore in accordance with the literature showing mostly normal patterns on pulmonary function tests. Especially in patients with thoracic dominant breathing with insufficient ventilation, it might be worthwhile considering an increased residual volume on body plethysmography as hint for an underlying functional respiratory disorder, but this can only be interpreted in conjunction with clinical findings, and the observed difference did not reach significance in our cohort. Whether these changes in breathing patterns might in part be caused by diagphragmal muscle weakness [10] or a part of a multifactorial genesis remains to be elucidated.

In the small cohort presented here, the diagnosis of functional respiratory disorders was based on a combination of clinical history regarding symptoms and mostly provocation of these symptoms and breathing patterns after standardized exercise testing. The Nijmegen questionnaire [29] in adults and the SHAPE questionnaire [30] in children as well as the hyperventilation provocation test [31] have been used to aid diagnosis of functional respiratory disorders, but none of the standardized tests has been helpful in diagnosing different subtypes of dysfunctional breathing [32]. Clinical evaluation of breathing patterns while resting and on exercise has been helpful to correctly diagnose functional respiratory disorders [33]. In conclusion, the approach presented in our cohort therefore seems reasonable for a real-world setting, especially if clinical history and examination are clearly congruent with a functional respiratory disorder.

Functional respiratory disorders [34] and long-COVID [6] have been more commonly diagnosed in teenage girls. Our cohort reports on a significant proportion of male teenagers with functional respiratory disorders. Even though we only report on a small cohort, it is important to consider functional respiratory disorders in all sexes.

All patients reported improvement of symptoms on follow-up after education about the benign nature of the condition and breathing exercises led by experienced physiotherapists. Breathing retraining with a combination of relaxation techniques and breathing exercises have been shown to be helpful in the treatment of functional respiratory disorders [35, 36]. In our cohort so far, only a small number of patients was followed up, but after 3 months, symptoms seem to improve with abovementioned therapeutic measures. It is important to personalize rebreathing training depending on the exact nature of the functional respiratory disorder identified. Patients with inducible laryngeal obstruction need more focus on the upper airways, whereas thoracic dominant breathing patterns with insufficient ventilation profit from retraining of abdominal/diaphragmal breathing.

### Limitations

Limitations of the study include the single-center setting and the retrospective design. Additionally, no cardiopulmonary exercise testing was performed but only treadmill exercises. No standardized tests as questionnaires or hyperventilation tests were applied but thorough clinical examination of breathing patterns while resting and while exercising, as well as after exercising by experienced pediatric pulmologists. Some of the patients had pre-diagnosed asthma which has been reported in a significant number of children with post-acute SARS-CoV-2 respiratory symptoms [17]. No PFT after treadmill exercise showed hints for bronchial obstruction or increased airway resistance; therefore, at least in our cohort, asthma seems to be not causal for the symptoms reported. Psychological involvement seems to be common in patients experiencing functional respiratory disorders in general [34] as well as after SARS-CoV-2 infection [21, 37]. The retrospective nature of the study did not allow to assess this aspect in detail in our cohort but should be considered in all patients with dysfunctional breathing.

## Conclusions

This study offers a pragmatic, real-world approach to evaluate children and adolescents with post-SARS-CoV-2 sequelae regarding pulmonary symptoms. Thorough history taking and a detailed clinical evaluation of breathing patterns when resting and exercising might save some patients invasive and cost-/time-intensive diagnostics. It is important to differentiate different types of functional respiratory disorders to optimally educate and train the patients regarding breathing exercises. The patients that were followed up all reported improvement of their symptoms. Therapeutic approaches should include detailed education about the benign nature of the condition and the absence of pulmonary pathology. Likewise, physiotherapy with breathing exercises can be successfully used to reestablish physiologic breathing patterns. Nonetheless, functional respiratory disorders should only be considered if other differential diagnoses, e.g., cardiac involvement, have been ruled out.

## Data Availability

Data are available upon reasonable request from the corresponding author.
